# ShangRing versus Mogen clamp for early infant male circumcision in eastern sub-Saharan Africa: a multicentre, non-inferiority, adaptive, randomised controlled trial

**DOI:** 10.1016/S2214-109X(22)00326-6

**Published:** 2022-09-13

**Authors:** Spyridon P Basourakos, Quincy G Nang, Karla V Ballman, Omar Al Hussein Al Awamlh, Nahid Punjani, Kaylee Ho, Mark A Barone, Quentin D Awori, Daniel Ouma, Jairus Oketch, Alice E Christensen, Augustino Hellar, Maende Makokha, Alphonce Isangu, Robert Salim, Jackson Lija, Ronald H Gray, Stephen Kiboneka, Aggrey Anok, Godfrey Kigozi, Regina Nakabuye, Charles Ddamulira, Andrew Mulooki, Silas Odiya, Rose Nazziwa, Marc Goldstein, Philip S Li, Richard K Lee

**Affiliations:** a Department of Urology, Weill Cornell Medicine, New York–Presbyterian Hospital, New York, NY, USA; b Division of Biostatistics, Department of Population Health Sciences, Weill Cornell Medicine, New York–Presbyterian Hospital, New York, NY, USA; c Center for Biomedical Research, Population Council, New York, NY, USA; d Population Council, Nairobi, Kenya; e Homa Bay Teaching and Referral Hospital, Nairobi, Kenya; f Jhpiego Tanzania, Dar es Salaam, Tanzania; g Ministry of Health, Tanzania; h Johns Hopkins University, Bloomberg School of Public Health, Baltimore, MD, USA; i Uganda Virus Research Institute, Entebbe, Uganda

## Abstract

**Background:**

Use of medical devices represents a unique opportunity to facilitate scale-up of early infant male circumcision (EIMC) across sub-Saharan Africa. The ShangRing, a circumcision device prequalified by WHO, is approved for use in adults and adolescents and requires topical anaesthesia only. We aimed to investigate the safety and efficacy of the ShangRing versus the Mogen clamp for EIMC in infants across eastern sub-Saharan Africa.

**Methods:**

In this multicentre, non-inferiority, open-label, randomised controlled trial, we enrolled healthy male infants (aged <60 days), with a gestational age of at least 37 weeks and a birthweight of at least 2·5 kg, from 11 community and referral centres in Kenya, Tanzania, and Uganda. Infants were randomly assigned (1:1) by a computer-generated text message service to undergo EIMC by either the ShangRing or the Mogen clamp. The primary endpoint was safety, defined as the number and severity of adverse events (AEs), analysed in the intention-to-treat population (all infants who underwent an EIMC procedure) with a non-inferiority margin of 2% for the difference in moderate and severe AEs. This trial is registered with Clinical. Trials.gov, NCT03338699, and is complete.

**Findings:**

Between Sept 17, 2018, and Dec 20, 2019, a total of 1420 infants were assessed for eligibility, of whom 1378 (97·0%) were enrolled. 689 (50·0%) infants were randomly assigned to undergo EIMC by ShangRing and 689 (50·0%) by Mogen clamp. 43 (6·2%) adverse events were observed in the ShangRing group and 61 (8·9%) in the Mogen clamp group (p=0·078). The most common treatment-related AE was intraoperative pain (Neonatal Infant Pain Scale score ≥5), with 19 (2·8%) events in the ShangRing and 23 (3·3%) in the Mogel clamp group. Rates of moderate and severe AEs were similar between both groups (29 [4·2%] in the ShangRing group *vs* 30 [4·4%] in the Mogen clamp group; difference –0·1%; one-sided 95% CI upper limit of 1·7%; p=0·89). No treatment-related deaths were reported.

**Interpretation:**

Use of the ShangRing device for EIMC showed safety, achieved high caregiver satisfaction, and did not differ from the Mogen clamp in other key measures. The ShangRing could be used by health systems and international organisations to further scale up EIMC across sub-Saharan Africa.

**Funding:**

Bill & Melinda Gates Foundation.

## Introduction

Observational studies suggested in 1986 that voluntary medical male circumcision (VMMC) was associated with reduced male HIV acquisition.^1^, ^2^ Three subsequent randomised controlled trials in sub-Saharan Africa showed that VMMC reduced HIV incidence in men by 50–60%.^3^, ^4^, ^5^ Furthermore, VMMC has been associated with a reduction in risk of urinary tract infections, phimosis, and balanitis in men,^6^, ^7^, ^8^, ^9^, ^10^, ^11^, ^12^ as well as reduction in risk of cervical cancer in female sexual partners and sexually transmitted infections in both men and women.^13^ WHO and UNAIDS recommend that countries with low rates of male circumcision and a high incidence of heterosexually transmitted HIV infections include VMMC as part of their comprehensive HIV prevention strategies.^14^

15 countries in east and southern Africa with low rates of VMMC and a high incidence of HIV have scaled up VMMC programmes, which resulted in nearly 27 million male circumcisions by the end of 2020.^15^, ^16^ The use of medical devices to perform VMMC has been proposed to facilitate and ultimately achieve WHO's goal of 90% access to VMMC by 2020, which has not been reached to date. Although the majority of VMMC procedures are performed in adolescent and adult men, the most long-term coverage and potential population-level impact on HIV transmission might ultimately be achieved through early infant male circumcision (EIMC). EIMC is simpler and has the benefit of more rapid wound healing compared with VMMC in adolescent boys and adult men, which simplifies postoperative care and avoids the risk of early resumption of sexual intercourse seen with older age groups. Additionally, some data have shown that EIMC could be safer and less expensive than standard VMMC.^17^

**Research in context**

**Evidence before this study**

We searched PubMed on Sept 1, 2018, before the initiation of the trial, for articles published in English only without date restrictions. Previous studies have shown that voluntary medical male circumcision (VMMC) reduces the incidence of HIV and urinary tract infections in men. Although most VMMC procedures are performed in adolescents and adults, the most long-term coverage and potential population-level impact on HIV transmission might ultimately be achieved through early infant male circumcision (EIMC). Medical devices simplify the performance of VMMC and, therefore, have been proposed to facilitate WHO's goal of 90% access to VMMC in sub-Saharan Africa by 2020, which has not been reached to date. The ShangRing device is the only medical circumcision device prequalified by WHO for use in individuals from age 10 years to adulthood; however, its use in EIMC has not been explored previously.

**Added value of this study**

In this multicentre, non-inferiority, open-label, adaptive randomised controlled trial in eastern sub-Saharan Africa, we compared the safety and efficacy of the ShangRing versus the Mogen clamp, which is currently considered to be the gold standard for EIMC, in a cohort of 1420 male infants. To our knowledge, this is the first randomised controlled trial to assess the use of ShangRing for EIMC in this age group. Our results show that EIMC by ShangRing is safe and offers excellent outcomes.

**Implications of all the available evidence**

Use of the ShangRing in EIMC showed safety and achieved high caregiver satisfaction. This device could be used by health systems and international organisations to further scale up EIMC efforts across sub-Saharan Africa.

Medical devices simplify the performance of VMMC and facilitate task shifting from physicians to non-physicians to augment service delivery. The ShangRing is the only male circumcision device prequalified by WHO for use in individuals from age 10 years to adulthood.^18^ We aimed to evaluate the safety and efficacy of the ShangRing versus the Mogen clamp for EIMC in infants across eastern sub-Saharan Africa.

## Methods

### Study design and participants

We conducted a multicentre, non-inferiority, open-label, randomised controlled trial in 11 community and referral centres across Kenya (Homa Bay and Kisumu), Tanzania (Ngome Health Center, Iringa Regional Hospital, Mafinga District Hospital, and Ilula Hospital), and Uganda (Kakuuto, Kalisizo, Lyantonde, Masaka, and Rakai). Eligible participants were healthy male infants (aged <60 days) with a gestational age of at least 37 weeks and a birthweight of at least 2·5 kg, who had been brought to the centres to discuss circumcision. Infants with perinatal illnesses requiring treatment, congenital genitourinary abnormality requiring surgical repair (eg, hypospadias), allergies to known anaesthetic components, or a family history of bleeding disorders were excluded.

The trial was reviewed and approved by the institutional review board at Weill Cornell Medicine (New York, NY, USA) and institutional review boards or medical councils from each collaborating organisation (Jhpiego, Johns Hopkins University, Baltimore, MD, USA) and participating country (Kenya, Tanzania, Uganda). Written informed consent was obtained from the parents or legally authorised representatives of infants at enrolment. The trial, data collection, and data management adhered to good clinical practice guidelines for clinical trials.

### Randomisation and masking

Enrolled infants were randomly assigned (1:1) to undergo EIMC by either the ShangRing or the Mogen clamp. Random allocation was achieved with a text message service (Sealed Envelope, London, UK). The allocation sequence was generated by computer, uploaded to Sealed Envelope, and entered into a secured electronic database. Parents and legally authorised representatives, investigators, physicians and non-physicians, and providers who evaluated infants for pain and other adverse events (AEs) after the procedure were unmasked to the allocated intervention.

### Procedures

All participating infants received a rectal suppository of 40 mg acetaminophen 30 mins before the procedure and a 2·0% sucrose solution as a pacifier during the procedure. A mixture of topical 2·5% lidocaine and 2·5% prilocaine cream was applied to the shaft and the glans of the penis before any intervention. Dwell time was measured from agent application to either the absence of response to a noxious stimulus on the foreskin or to the start of the procedure. Supplemental injectable local anaesthetic was available if inadequate analgesia was noted by the provider. The Neonatal Infant Pain Scale (NIPS) score was used to standardise pain assessment. NIPS is a validated pain scale assigning points on the basis of the provider's observation of the infant's facial expression, cry, breathing pattern, arms, legs, and state of arousal.^19^ NIPS scores range from zero to ten, with a higher score correlating with higher probability of more severe pain.

**Figure 1:**
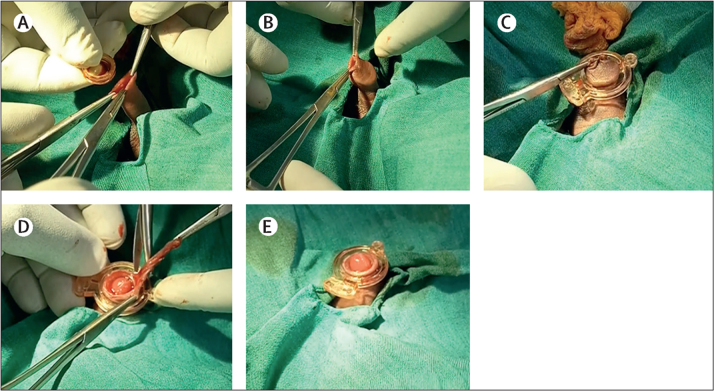
Procedural steps of ShangRing procedure for EIMC A) Insertion of the inner ring under the foreskin. B) Appropriate position of the inner ring. C) Application of the outer ring. D) Removal of the foreskin trapped between the two rings with scissors. E) Completion of the circumcision with the outer ring in place. EIMC=early infant male circumcision.

Before trial initiation, all staff performing the procedure completed a training course on EIMC using both devices. The previously described no-flip technique^20^ was used for all infants randomly assigned to undergo circumcision by ShangRing. First, the foreskin and glans penis were separated. Second, the inner ring was inserted and adjusted to encircle the glans and frenulum (a dorsal slit was performed to facilitate this step when necessary). Third, the outer ring was affixed to the inner ring on the outside to compress the foreskin. Fourth, the excess foreskin distal to the rings was removed, leaving the device in place (figure 1). Finally, the wound was dressed and the family was instructed that the device would either fall off on its own within 1–2 weeks or be removed by the surgical team after postoperative day 7. The Mogen clamp procedure was performed as previously described.^21^ After the procedure, infants were monitored for immediate complications according to standard protocols at each site. A systematic pain assessment was conducted approximately 20 mins after the procedure, and a formal wound assessment was performed before hospital discharge. Parents and legally authorised representatives were given wound care instructions and a phone number to contact a provider for any questions or concerns 24 h a day, 7 days a week. The cadre of 1378 providers comprised nurses (1068 [77·5%]), clinical officers (303 [22·0%]), and doctors (seven [0·5%]).

Follow-up was performed by an initial telephone call to parents or legally authorised representatives 24 h after the procedure, followed by scheduled clinic visits every 7 days until complete wound healing. Complete healing was defined as complete re-epithelialisation of the circumcision incision. Infants whose parents or legally authorised representatives could not be reached despite multiple telephone attempts were seen in person at the follow-up visits. Interviews assessing satisfaction among parents and legally authorised representatives were conducted at the last follow-up visit.

### Outcomes

The primary endpoint was safety, defined as the number and severity of AEs. AEs were prespecified as mild, moderate, or severe on the basis of WHO definitions (appendix p 62).^22^ The study teams were responsible for the clinical management and follow-up of all reported AEs. Principal investigators and staff from each study site conducted a daily review of AEs to determine their severity and their association with the circumcision procedure. All serious AEs were reported to the principal investigators, sponsors, and institutional review boards.

Secondary endpoints included time to complete wound healing; the ease of use associated with both circumcision devices and provider preference, determined by procedure time and problems encountered during the procedure provided by in postoperative interviews; neonatal infant pain scale scores, assessed by NIPS; and satisfaction among parents and legally authorised representatives, evaluated by interviews at the final follow-up visit.

### Statistical analysis

Sample size was estimated using WHO's requirements for prequalification of adult and adolescent circumcision devices. We expected that the benefits of the ShangRing would allow a non-inferiority margin of 2·0% for the AE rate. This margin was selected because the anticipated rate of moderate and severe AEs is 2·0% for the Mogen clamp based on the literature.^21^, ^23^ A sample size of at least 1200 eligible infants (600 per intervention group) with one formal interim analysis for futility with 80% power and one-sided alpha of 0·05 was deemed necessary. An interim analysis for futility was conducted after the first 100 infants were enrolled to assess the rate of moderate and severe AEs. The sample size was not adjusted for this analysis because there was minimal effect on the trial operating characteristics. The trial would be stopped if the Z score was 1·91 or greater. In addition, the trial would be stopped if notably higher rates of severe or serious AEs occurred than have been seen in previous studies on EIMC in Africa, or if the observed rate of moderate and severe AEs exceeded 5·0%. There were no bias adjustments planned for the interim analysis. The interim analysis was conducted by the study statisticians (KH and KVB). The statistical analysis plan can be found in the appendix (pp 3–61). The interim analysis was reviewed by a data and safety monitoring board comprised three members.

The primary endpoint was analysed in the intention-to-treat population, defined as all infants who underwent an EIMC procedure. We established non-inferiority in the rate of moderate and severe AEs for use of ShangRing versus use of the Mogen clamp using a one-sided 95% Agresti-Caffo^24^ CI and ascertaining whether it contained 2·0%. As a secondary analysis of the primary endpoint, a bootstrap method (5000 bootstrap samples) was used to generate the one-sided 95% CI to account for potential within site correlations. Continuous data are summarised as mean (SD) or median (IQR), and categorical data are summarised as frequency (%). Cumulative distributions were established for time to complete wound healing. We used a two-sample *t* test and Kaplan-Meier survival analysis using a log-rank test to compare the time to complete wound healing between the two intervention groups. In the event that a participant received an intervention that differed from the one randomly assigned, the discrepancy was documented and an intention-to-treat analysis was performed. Continuous variables were analysed with the Student's *t* test and categorical variables with the χ^2^ test (including a version that generated exact p values). Differences in proportions are reported as a point estimate and a 95% Agresti-Caffo CI. Statistical significance was set at p=0·05 and all tests were two-sided for secondary analyses. The p values were not adjusted for the multiple secondary analyses. Statistical analyses were done with R (version 3.6.1). This trial is registered with Clinical. Trials.gov, NCT03338699.

### Role of the funding source

The funder of the study had no role in study design, data collection, data analysis, data interpretation, or writing of the report.

## Results

**Figure 2:**
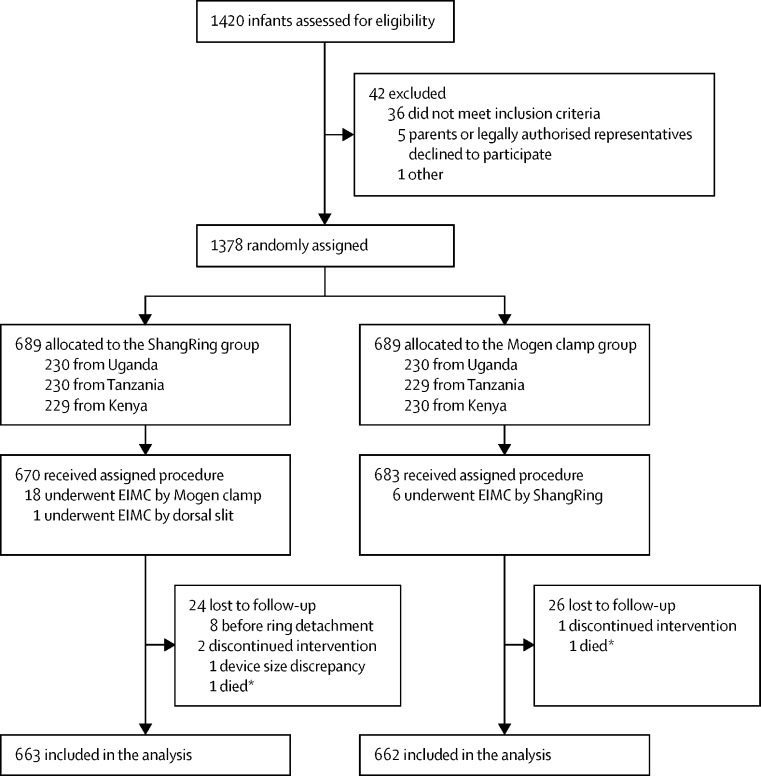
Trial profile EIMC=early infant male circumcision. *Deemed to be unrelated to the EIMC procedure.

Between Sept 17, 2018, and Dec 20, 2019, a total of 1420 infants were assessed for eligibility, of whom 1378 (97·0%) were enrolled. 689 (50·0%) infants were randomly assigned to undergo EIMC by ShangRing and 689 (50·0%) by Mogen clamp (figure 2). The median age of infants was 24·0 days (IQR 11·0–43·0) in the ShangRing group and 25·0 days (12·0–42·0) in the Mogen clamp group (table 1). Among parents and legally authorised representatives, median age was 27·0 years in both the ShangRing group (IQR 22·0–32·0) and the Mogen clamp group (23·0–32·0), and most representatives (1301 [94·4%] of 1378) were the infant's mother. Primary motivations for parents or legally authorised representatives to seek EIMC for their infant were disease prevention, hygiene, and social or religious reasons (table 1). The highest attained educational levels of parents or legally authorised representatives ranged from none to postdoctoral studies. Additional demographic characteristics between both intervention groups were well balanced (table 1).

**Table 1:** Baseline characteristics of infants and parents or legally authorised representatives

			**ShangRing (n=689)**	**Mogen clamp (n=689)**
Age, days			24·0 (11·0–43·0)	25·0 (12·0–42·0)
Weight, kg			4·1 (3·5–4·7)	4·1 (3·5–4·9)
Age of parent or legally authorised representatives, years			27·0 (22·0–32·0)	27·0 (23·0–32·0)
Ethinic group of parent or legally authorised representatives				
	Luo		202 (29·3%)	200 (29·0%)
	Baganda		164 (23·8%)	164 (23·8%)
	Hehe		121 (17·6%)	107 (15·5%)
	Banyankole		37 (5·4%)	38 (5·5%)
	Bena		32 (4·6%)	37 (5·4%)
	Luhya		17 (2·5%)	14 (2·0%)
	Wakinga		12 (1·7%)	15 (2·2%)
	Kisii		6 (0·9%)	7 (1·0%)
	Other		95 (13·8%)	105 (15·2%)
	Data missing		3 (0·4%)	2 (0·3%)
Mode of infant delivery				
	Spontaneous vaginal delivery		538 (78·1%)	522 (75·8%)
	Caesarean section		151 (21·9%)	167 (24·2%)
Country and site				
	Uganda		230 (33·4%)	230 (33·4%)
		Kakuuto	56 (24·3%)	43 (19%)
		Kalisizo	31 (13·5%)	36 (16%)
		Lyantonde	26 (11·3%)	29 (13%)
		Masaka	57 (24·8%)	70 (30%)
		Rakai	60 (26·1%)	52 (23%)
	Tanzania		230 (33·4%)	229 (33·2%)
		Ngome Health Center	46 (20·0%)	55 (24·0%)
		Iringa Regional Hospital	56 (24·3%)	58 (25·3%)
		Mafinga District Hospital	75 (32·6%)	76 (33·2%)
		Ilula Hospital	53 (23·0%)	40 (17·5%)
	Kenya		229 (33·2%)	230 (33·4%)
		Homa Bay	112 (48·9%)	118 (51·3%)
		Kisumu	117 (51·1%)	112 (48·7%)
Motivation for parent or legally authorised representatives to seek EIMC for infant				
		Disease protection (eg, HIV and other STIs)	447 (64·9%)	444 (64·4%)
Hygiene			197 (28·6%)	212 (30·8%)
Social or religious reason			29 (4·2%)	21 (3·0%)
As part of medical therapy			10 (1·5%)	9 (1·3%)
Other			3 (0·4%)	1 (0·1%)
Data missing			3 (0·4%)	2 (0·3%)

Data are median (IQR) or n (%). EIMC=early infant male circumcision. STI=sexually transmitted infection.

Of the 689 infants randomly assigned to undergo EIMC by ShangRing, 670 (97·2%) received their assigned procedure while 18 (2·6%) underwent EIMC with the Mogen clamp and one (0·1%) underwent EIMC by dorsal slit alone (figure 2). Of the 689 infants randomly assigned to undergo EIMC by Mogen clamp, 683 (99·1%) received their assigned treatment while the remaining six (0·9%) underwent EIMC by ShangRing (difference –1·9% [95% CI –3·3% to –0·4%]). Reasons for protocol violation included the inability to find an appropriately sized ring to accommodate the size of the penis among 14 (2·0%) infants in the ShangRing group, clerical errors by study personnel in six (0·9%) infants in the ShangRing group and four (0·6%) infants in the Mogen clamp group, and ineligibility for clinical reasons in one (0·1%) infant in the ShangRing group. Of the 670 infants in the ShangRing group to receive the assigned procedure, a mean foreskin slit of 7 mm (SD 3 mm) was required to facilitate insertion of the inner ring in 552 (82·4%; [95% CI 77% to 83%]) infants. There was no significant difference in the mean time required to complete the procedures (12·3 mins [SD 8·0] in the ShangRing group *vs* 12·4 mins [9·5] in the Mogen clamp group; mean difference –0·1 mins [95% CI –1·0 to 0·8]). In both groups, the procedures were performed mainly by nurses, followed by clinical officers and doctors (table 2). All providers were trained in a minimum of five EIMC procedures by ShangRing before the start of the study.

**Table 2:** Perioperative characteristics of infants

		**ShangRing (n=689)**	**Mogen clamp (n=689)**	**p value**
Procedure duration, mins		12·3 (8·0)	12·4 (9·5)	0·85
Type of anaesthesia				
	Topical cream alone	669 (97·1%)	668 (97·0%)	1·0
	Topical cream with injectable	20 (2·9%)	21 (3·0%)	..
Anaesthetic dwell time, mins		57·5 (18·6)	58·9 (19·6)	0·17
Type of circumcision procedure conducted				
	ShangRing	670 (97·2%)	6 (0·9%)	<0·0001
	Mogen clamp	18 (2·6%)	683 (99·1%)	..
	Dorsal slit	1 (0·1%)	0	..
Cadre of provider performing circumcision				
	Nurse	524 (76·1%)	544 (79·0%)	0·35
	Clinical Officer	162 (23·5%)	141 (20·5%)	..
	Doctor	3 (0·4%)	4 (0·6%)	..
Cadre of provider assisting circumcision				
	Nurse	546 (79·2%)	538 (78·1%)	0·34
	Clinical Officer	129 (18·7%)	142 (20·6%)	..
	Doctor	14 (2·0%)	9 (1·3%)	..
Postoperative NIPS score*		0·3 (0·8)	0·3 (0·7)	0·29
Severe NIPS score (≥5)		19 (2·8%)	23 (3·3%)	0·66
Alleviation required beyond enteral acetaminophen		0	0	..
Wound status before discharge				
	Normal	684 (99·3%)	686 (99·6%)	1·0
	Data missing	2 (0·3%)	3 (0·4%)	..
ShangRing only		674 (97·8%)	NA	..
ShangRing size				
	V (15 mm)	1 (0·1%)	NA	..
	W (14 mm)	1 (0·1%)	NA	..
	X (13 mm)	100 (14·8%)	NA	..
	Y (12 mm)	193 (28·6%)	NA	..
	Z (11 mm)	379 (56·2%)	NA	..
Need for dorsal slit in the foreskin to insert ring		552 (81·9%)	NA	..

Data are mean (SD) or n (%). NIPS=Neonatal Infant Pain Scale. NA=not applicable.

* NIPS score was assessed approximately 20 mins after the procedure.

There were no missing data for the primary endpoint. There were few missing data for other endpoints and the analyses were done on a complete-case basis. A total of 43 (6·2%) AEs were observed in the ShangRing group and 61 (8·9%) in the Mogen clamp group, with no significant difference between both groups (p=0·078). We observed a total of 59 moderate and severe AEs, with no difference between both groups: 29 (4·2%) in the ShangRing group and 30 (4·4%) in the Mogen clamp group (difference 0·1%; one-sided 95% CI upper limit of 1·7%; p=0·89; table 3). Given that the upper limit is less than the non-inferiority margin of 2·0%, it can be assumed that ShangRing was non-inferior to the Mogen clamp with respect to the rate of moderate and severe AEs. The upper limit of the one-sided 95% CI was 0·3% based on the bootstrap method and 1·8% based on the per-protocol analysis, also less than 2·0%. There were nine (1·3%) moderate or severe AEs not related to pain in the ShangRing group and seven (1·0%) in the Mogen clamp group (difference 0·3% [95% CI –0·9% to 1·5%]). Two participants, one in the ShangRing group (0·1%) and one in Mogen clamp (0·1%), had two AEs each. One case of excessive foreskin removal and another case of insufficient foreskin removal were reported as AEs of moderate severity in the ShangRing group (table 3). No cases of glandular injury (partial or total transection), severe wound infection, or haemorrhage were recorded. Four serious AEs were reported following the procedure, two in each treatment group: one episode of jaundice in an infant in the ShangRing group (0·1%), an episode of sepsis in the Mogen clamp group (0·1%), and one death in both the ShangRing group (0·1%) and in the Mogen clamp group (0·1%) secondary to severe pneumonia. None of these AEs were deemed to be related to either of the EIMC procedures by the data and safety monitoring boards and respective institutional review boards.

**Table 3:** AEs among infants

			**ShangRing (n=689)**	**Mogen clamp (n=689)**
Total number			43 (6·2%)	61 (8·9%)
Number of moderate and severe AEs			29 (4·2%)	30 (4·4%)
Severity				
	Mild		12 (27·9%)*	29 (47·5%)†
	Moderate		6 (14·0%)	6 (9·8%)
		Bleeding	0	1 (1·6%)
		Excess skin removal	1 (2·3%)‡	0
		Inguinal hernia	1 (2·3%)	0
		Infection	1 (2·3%)	0
		Insufficient skin removal	1 (2·3%)§	0
		Adhesion	1 (2·3%)	2 (3·3%)
		Fever	1 (2·3%)	1 (1·6%)
		Rash	0	2 (3·3%)
	Severe		23 (53·5%)	24 (39·3%)
		Intraoperative NIPS score (≥5)	19 (44·2%)	23 (37·7%)
		Follow-up NIPS score (≥5)	3 (7·0%)	0
		Severe infection	0	0
		Glans transections	0	0
		Haemorrhage	0	0
		Neonatal sepsis	0	1 (1·6%)
		Jaundice	1 (2·3%)	0
	Serious		2 (4·7%)	2 (3·3%)
		Neonatal sepsis	0	1 (1·6%)
		Jaundice	1 (2·3%)	0
		Death¶	1 (2·3%)	1 (1·6%)

Data are n (%). AEs were prespecified as mild, moderate, severe, or serious on the basis of WHO definitions (appendix p 62). AE=adverse event. NIPS=Neonatal Infant Pain Scale.

* One (2·3%) infant from Uganda had two mild AEs: one after the procedure (bleeding) and one during follow-up (ulcer).

† One (2·3%) infant from Uganda had two mild AEs: one at 7-day follow-up (adhesion) and one at 14-day follow-up (ulcer).

‡ This case was managed conservatively with skin care.

§ This case was managed at the discretion of the provider and parent or legally authorised representatives.

¶ Occurred at postoperative day 30 in the ShangRing group and postoperative day 9 in the Mogen clamp group. Both causes of death were unknown but were deemed to be unrelated to the circumcision procedures by physicians.

Topical anaesthetic alone was successfully used for almost all EIMC procedures in both groups (difference 0·0% [95% CI –1·6% to 2·0%]; table 2). Mean anaesthetic dwell times were similar between both groups (–1·4 [–3·4 to 0·6]; table 2). Additional injectable anaesthetic was administered to the same proportion of infants in each group (0·0% [–2·0% to 1·7%]; table 2). The mean NIPS score 20 mins after the procedure did not differ between the two groups (0·0 mins [–0·1 to 0·1]; table 2). All severe pain-related AEs (NIPS scores ≥5) occurred postoperatively and did not significantly differ between the two techniques (–0·6% [–2·4% to 1·3%]). No medication outside of preoperative enteral acetaminophen was necessary to manage postoperative discomfort in either intervention group. Severe NIPS scores were reported in three (0·4%) infants who underwent EIMC by ShangRing during the follow-up period, compared with none of the infants who underwent EIMC by Mogen clamp.

**Figure 3:**
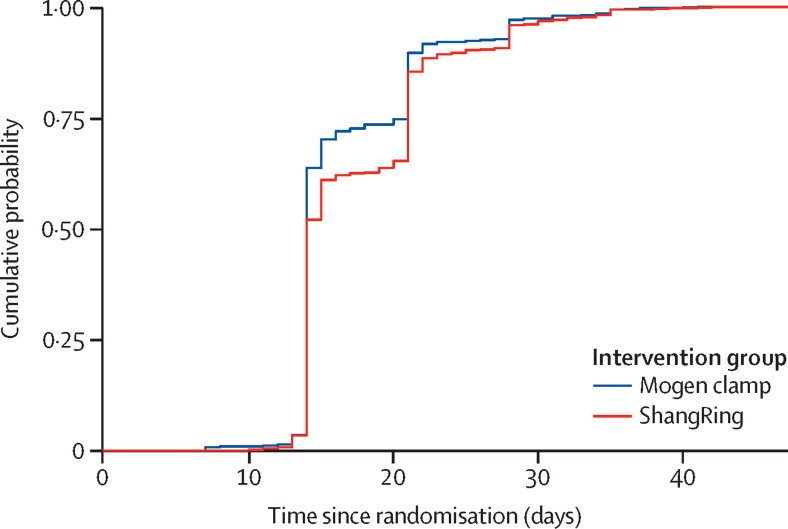
Kaplan-Meier curve of the cumulative probability of complete healing by intervention group

The mean time to complete healing was 18·0 days (SD 6·4) in the ShangRing group and 16·9 days (5·9) in the Mogen clamp group (difference 1·1 days [95% CI 0·4 to 1·8]; p=0·0009), favouring EIMC by Mogen clamp. By day 35, complete healing was observed in almost all infants (figure 3). In the ShangRing group, the ring need to be removed prematurely in four (0·6%) infants: for pain in three (0·4%) and swelling in one (0·1%).

A total of 1327 (96·3%; 95% CI 95·2% to 97·2%) parents and legally authorised representatives completed a satisfaction interview; 665 (96·5%) parents or legally authorised representatives of infants who underwent EIMC by ShangRing and 662 (96·1%) parents or legally authorised representatives of infants who underwent EIMC by Mogen clamp (difference 0·4% [95% CI –1·6% to 2·5%]). When parents and legally authorised representatives were asked what they liked about the procedure, top responses included less pain than expected, improved hygiene, cosmetic appearance, and short procedure time (table 4). When asked what they disliked about the procedure, most (1172 [88·3%] overall [95% CI 86·5% to 90·0%]) reported nothing. A minority of caregivers expressed concerns with pain, wound care, cosmesis, and healing times (table 4). When queried about the final appearance of the healed circumcised penis, 659 (99·0%) parents and legally authorised representatives of infants in the ShangRing group and 659 (99·5%) parents and legally authorised representatives of infants in the Mogen clamp group were satisfied (difference –0·4% [–1·4% to 0·5%]). Regardless of the circumcision method, 1319 (99·4%; 95% CI 99·2% to 99·8%) of 1327 parents and legally authorised representatives in total (659 [99·1%] of 665 in the ShangRing group and 660 [99·7%] of 662 in the Mogen clamp group) expressed that they would recommend EIMC to another parent or legally authorised representative.

**Table 4:** Satisfaction of parent or legally authorised representatives

	**ShangRing (n=665)**	**Mogen clamp (n=662)**	**p value**
Why they liked the EIMC procedure*			
Less pain than expected	278 (41·8%)	295 (44·6%)	0·36
Improved personal hygiene	277 (41·7%)	265 (39·8%)	0·55
Speed of procedure	254 (38·2%)	227 (34·3%)	0·14
Cosmetic appearance	242 (36·4%)	261 (39·4%)	0·30
No dressing applied to wound	202 (30·4%)	90 (13·6%)	<0·001
The device fell off on its own	194 (29·2%)	7 (1·1%)	<0·001
No stitches needed	157 (23·6%)	197 (29·8%)	0·015
No injection needed	142 (21·4%)	162 (24·5%)	0·21
Nothing	28 (4·2%)	31 (4·7%)	0·79
Why they disliked the EIMC procedure*			
Nothing	576 (86·6%)	596 (90·0%)	0·086
More pain than expected	20 (3·0%)	11 (1·7%)	0·18
Not the best cosmetic appearance	14 (2·1%)	2 (0·3%)	0·006
Wound care during healing was difficult	12 (1·8%)	20 (3·0%)	0·21
Circumcision took a long time to heal	8 (1·2%)	5 (0·8%)	0·58
**Satisfaction with the appearance of the healed circumcised penis**			
Satisfied	659 (99·1%)	659 (99·5%)	0·62
Dissatisfied	3 (0·5%)	1 (0·2%)	..
Data missing	3 (0·5%)	2 (0·3%)	..

Data are n (%). EIMC=early infant male circumcision.

* Multiple responses possible.

## Discussion

This is the first multicountry randomised controlled trial comparing the use of ShangRing versus Mogen clamp for EIMC in three eastern sub-Saharan countries. Our results show that the ShangRing was safe and efficacious for EIMC by non-physicians. AEs, postoperative pain, and satisfaction rates were similar between the two techniques. Thus, we believe that ShangRing could be included in the armamentarium of countries implementing EIMC and could help to achieve the goal of increasing the number of circumcisions and decreasing HIV transmission in Africa.

Except for pain, no severe or serious AEs related to the ShangRing were observed in this study. Importantly, severe complications, such as complete or partial transection of the glans, wound infection, or necrosis, which have been previously reported with the Mogen clamp and other devices, were not noted.^23^, ^25^ Additionally, the ShangRing showed lower rates of moderate AEs than did other medical devices used in male infants. The rate of moderate AEs in a study of 704 infants undergoing EIMC with an atraumatic circumcision device (AccuCirc, Clinical Innovations, Murray, UT, USA) was 2·8%, compared with a rate of 0·9% with the ShangRing.^26^ Although both the ShangRing and the Mogen clamp exceeded the expected 2·0% complication rate in this trial, when we limited our analysis to AEs that were definitely or probably related to the procedure, the percentage of AEs not related to pain was less than 1·0%. The absence of these severe complications might further reduce the barrier to EIMC uptake.^27^, ^28^ The majority of AEs were mild in nature, and most were observed in the Mogen clamp group. All AEs were managed conservatively and had no long-term consequences.

Pain was not only the most frequently reported but also the most challenging AE to assess in infants. We chose to use NIPS to minimise providers’ subjectivity;^29^ however, this scale has only been validated for assessing acute pain in neonatal intensive care units and not in surgical settings. Although most of the severe AEs were due to an intraoperative NIPS score above 5, none of these infants required further analgesic intervention. Thus, use of NIPS for intraoperative pain assessment seemed to be suboptimal. This limitation affected the ability to assess pain in infants undergoing EIMC by ShangRing or Mogen clamp. As an analogy, pain in adolescents and adults is only considered to be severe if it requires parenteral analgesia or leads to severe disability, yet this was not the case when pain was assessed with NIPS.^22^ Multimodal analgesia regimens have been deemed to be most effective in neonates and can include local topical anaesthetic with adequate dwell times (<80 mins), preoperative acetaminophen, adjunct methods (eg, sucrose pacifiers or music), and local injectable anaesthetic agents if adequate pain control is otherwise not achieved.^29^ In this trial, we differentiated our pain control approach from that recommended by WHO's injectable local anaesthesia regimen.^30^ We preferred to use an acetaminophen rectal suppository plus topical anaesthetic because of greater ease of use by non-medical personnel and a potentially lower risk of complications than injectable anaesthetic agents. Even though more than 97% of the EIMC procedures by ShangRing were successfully performed in infants with only local topical anaesthetics, we recommend that injectable anaesthetic agents are available for redundancy. Furthermore, providers who use local anaesthetics need to familiarise themselves with all of the precautions for safe use of these medications.^30^ For example, clinicians might need to be aware of excess absorption through unintended application or spreading of anaesthetic to other exposed areas of skin.

Both the ShangRing and Mogen clamp techniques showed short and similar procedural times and were easily performed by non-physicians. Notably, all physicians, nurses, and clinical officers safely performed both interventions, similar to rates of successful medical circumcision interventions in older individuals. Several studies have found no association between AEs and provider type, which further facilitates task-shifting initiatives.^31^ To better understand shortcomings related to the ShangRing technique, closer attention was paid to the reasons why infants were transitioned intraoperatively to undergo EIMC by Mogen clamp instead, despite being randomly assigned to EIMC by ShangRing at the start of the study. We found that crossover in this group of infants mostly occurred secondary to the absence of an appropriately sized ShangRing. Use of a ShangRing device that does not fit adequately has been associated with negative outcomes. Fang and colleagues^32^ proposed that the smaller the difference between the ShangRing size and glans diameter, the lower the presence of postoperative oedema and foreskin asymmetry. The smallest size of ShangRing available to investigators in this trial was size Z, with the inner diameter of the inner ring measuring 11 mm.

We also explored the effects of EIMC on parents and legally authorised representatives. The newborn and early infant period is a stressful time for caregivers. Therefore, maximising the satisfaction of parents and legally authorised representatives and understanding their motivations behind opting their infant for EIMC are of paramount importance in all efforts to scale up EIMC. Mavhu and colleagues^33^ explored these motivations among 247 families in Zimbabwe through focus groups, and found that mothers had a crucial role in the decision-making process. Similarly, mothers in the current study represented the majority of legally authorised representatives. Parents and legally authorised representatives were mostly satisfied with the experience and reported hygiene and disease protection as their primary motivation behind opting their infant for EIMC, probably due to the impact of ongoing VMMC education efforts in sub-Saharan Africa.^28^ Additionally, less than 1·0% of parents and legally authorised representatives were not satisfied with the appearance of the infant's healed circumcised penis.

This study has several limitations. First, the use of multiple sites and personnel introduces heterogeneity in terms of interpretation of clinical data, even though it improves the generalisability of our findings. Second, the trial was designed to detect non-inferiority of the ShangRing versus Mogen clamp, a widely used device for EIMC. Our results suggest that the two techniques do not differ in efficacy, usability, or, most importantly, their safety profile. Third, the NIPS was not as effective at sufficiently differentiating between mild, moderate, and severe pain in infants as has been reported in adolescent and adults. Fourth, paternal circumcision status was not obtained when assessing the views of parents and legally authorised representatives on EIMC, despite its previously described influence on perceptions of EIMC.^28^ Future efforts are needed to better characterise caregivers’ motivations behind opting their infant for EIMC. Fifth, there was no adjustment for bias as the result of the interim analysis. However, the amount of bias would be minimal due to the small number of patients in the interim analysis and because the interim analysis was done to assess futility only. Additionally, we acknowledge that potential bias might exist due to the unmasked nature of providers who evaluated participants for pain and other AEs after circumcision. Finally, considerations around availability of resources and supply of devices were not addressed in this trial. Although a cost analysis of this trial is ongoing, implementing scale-up of EIMC by use of a non-reusable device such as the ShangRing will probably require added costs initially. However, potential use of a single device across all age groups is compelling, in part because of the possible cost-saving implications that would result from increased uptake across multiple age groups (eg, simplified supply chain and streamlined provider training). Further longitudinal studies will be needed to show these benefits.

EIMC by the ShangRing is non-inferior to EIMC by the Mogen clamp and represents a safe and efficacious tool with excellent outcomes and a mostly high satisfaction rate among parents and legally authorised representatives. Despite how accurate pain evaluation was challenging in this trial, almost all EIMC procedures by ShangRing were successfully performed with use of local topical anaesthetics only. Thus, this device could be used by health systems and international organisations as an integral part of efforts to scale up future EIMC services across sub-Saharan Africa.

## Data sharing

Data collected for the study, including individual participant data and a data dictionary defining each field in the set, will be made available to the sponsor and participating researchers. Deidentified patient data, the data dictionary, the study protocol, and the informed consent form will be made available at publication upon request to the corresponding author with investigator approval and a signed data access agreement.

## Declaration of interests

We declare no competing interests.
