# Supplementary material for: ShangRing versus Mogen clamp for early infant male circumcision in eastern sub-Saharan Africa: a multicentre, non-inferiority, adaptive, randomised controlled trial
Source: Lancet Glob Health. 2022 Sep 13;10(10):e1514–22. doi: 10.1016/S2214-109X(22)00326-6 (PMC9638032; doi:10.1016/S2214-109X(22)00326-6)
Supplement: Supplementary appendix [file mmc1.pdf]

THE LANCET
Global Health

Supplementary appendix

This appendix formed part of the original submission and has been peer reviewed. We post it as supplied by the authors.

Supplement to: Basourakos SP, Nang QG, Ballman KV, et al. ShangRing versus Mogen clamp for early infant male circumcision in eastern sub-Saharan Africa: a multicentre, non-inferiority, adaptive, randomised controlled trial. *Lancet Glob Health* 2022;
10: e1514–22.

ShangRing versus Mogen clamp for early infant male circumcision in eastern sub-Saharan Africa: a multicentre, non-inferiority, adaptive, randomised controlled trial

Supplementary Material

Evaluation of the ShangRing vs. Mogen Clamp for Early Infant Male Circumcision in sub-Saharan Africa

Statistical Analysis Plan

Phase I

Safety and Acceptability of the no-flip ShangRing Technique vs. Mogen Clamp

Sponsors: **Weill Cornell Medicine**
525 E. 68th Street
New York, NY 10065, USA

Funded By: **The Bill & Melinda Gates Foundation**

Table of Contents

1.0 .... Study summary 3

1.1 Objectives & Endpoints for Phase 1 3

1.2 Sample Size Justification 4

1.3 Electronic Case Reporting Forms 4

1.4 Data collection using the eCRFs 5

1.5 Data confidentiality 6

1.6 Data backup 6

1.7 Summary of Analysis Strategy 6

2.0 .... Final analysis table shells 9

2.1 Enrollment & final status 9

2.2 Demographic Information 13

2.3 Intake 17

2.4 Eligibility for Circumcision & Study 21

2.5 Operation 23

2.6 Post Operation 32

2.7 Follow-up 34

2.8 Healing information 41

2.9 Completion Interview 42

2.10 Adverse Events 45

1.0 Study summary

The study is to evaluate the safety effectiveness and acceptability of the ShangRing device for early infant male circumcision (EIMC). The study will be conducted in two phases:

- Phase 1 is to randomize approximately 1360 eligible infants 1:1 to receive either ShangRing or Mogen Clamp circumcision. The Mogen Clamp is one of the EIMC devices included in the WHO training materials for EIMC and is considered the control procedure for this trial. Circumcisions will be conducted by physicians and non-physicians. Assessments of interest are circumcision time, pain, adverse events (AEs), healing course, and parent and provider preferences.

- Phase 2 is a non-comparative field study and will enroll approximately 1800 eligible infants who will undergo ShangRing EIMC performed in routine settings. Surgical outcomes will be similar to the first phase. This is a non-comparative field study.

Participants will be enrolled at 12 sites across Kenya(x3), Tanzania(x4), and Uganda(x5). The study population will include male infants ≤60 days old.

The study being conducted by Weill Cornell Medicine in conjunction with the Population Council. , Jhpiego, the Rakai Health Sciences Program, Johns Hopkins University, George Washington University, and the Uganda Virus Research Institute.

This Statistical Analysis Plan is for Phase 1 of the study. Another data analysis plan will be developed for Phase 2.

1.1 Objectives & Endpoints for Phase 1

1.1.1 Primary objective and endpoints

Evaluate the safety and acceptability of the no-flip ShangRing Technique vs, Mogen clamp for EIMC in patients up to 60 days old when used by physicians and non-physicians. The primary endpoint is the rate of AEs following EIMC conducted by physicians and medical providers (non-physician). Other safety endpoints include documentation of time to complete clinical wound healing, device-related malfunctions, and occurrence of device displacement.

1.1.2 Secondary Objectives and endpoints

- Assess ease of use of the ShangRing for EIMC, which will be determined by procedure time and problems encountered during the procedure

- Evaluate satisfaction of ShangRing EIMC among parents of study participants, which will be determined by parent interviews to document acceptability and satisfaction with the ShangRing circumcision as well as procedure and post-procedure pain

- Evaluate satisfaction of using the ShangRing for EIMC among VMMC providers-to document experiences with the use of the ShangRing, problems encountered during medical procedures, and preference of VMMC technique

- Determine the occurrence and safety of spontaneous detachment, which will be determined with data on the timing of spontaneous detachment and documentation of AEs and time course of complete

clinical would healing at clinical examination, as well as problems/complaints reported during patient interviews

1.2 Sample Size Justification

The goal is to determine if the AE rate using the ShangRing is not inferior to that for the control (i.e., the Mogen clamp). The anticipated moderate-severe AE rate is 2% for the Mogen clamp control arm. We expect that the benefits of the ShangRing in terms of convenience and acceptability will allow a non-inferiority margin of 2% (absolute). The total sample size combining all three countries of at least 1200 evaluable infants (600 per arm), with one formal interim analysis for futility has approximately 80% power with a (one-sided) alpha = 0.05. The null hypothesis of the non-inferiority test is that the moderate/adverse AE rate for the ShangRing is at least 2% (absolute) higher than that of Mogen clamp, which is assumed to be 2%. The alternative hypothesis is that the moderate/severe adverse event rate of the ShangRing is less than 2% greater than that of the Mogen clamp.

1.3 Electronic Case Reporting Forms

The first stage of data management is the development of the electronic case report forms (eCRFs) using finalized word versions of the case report forms. A total of 10 eCRFs have been developed to capture a wide range of information as follows:

1. Screening visit (DAY 0-SCREEN): This form records screening information and responses to inclusion/exclusion criteria for the study. This form is filled on Day 0

2. Participant demographic data (DAY 0-DEMO): This form records demographic data of the participants. This form is filled on Day 0

3. History and examination (DAY 0-INTAKE): This form collects the medical history of the participant and includes the screening questions to determine eligibility for the study and VMMC. This form is filled on Day 0

4. Operation (DAY 0-OPR): This form gives a record of the operation procedures in the circumcision process and the time taken to perform the surgery. It included information on the participant’s random assignment. This form is filled on Day 0

5. Master participant follow-up form (MPFU): This form is used to gather information during all follow-up visits (scheduled and unscheduled), including information on the removal, status of the wound, and any symptoms of the adverse event. This form includes information on AE, SAEs, and concomitant medication. This form is filled on day 7 and beyond.

6. Post-operation (DAY 0-POST-OPR): This form is used to gather information during the post-op period, including any AEs or medications given.

7. 24hr Call Interview: This form provides information about the status of the infant on POD 1 via phone call to parent/LAR.

8. Completion interview (IVW): This form is used during the interview at the final follow-up visit, which occurs when the participant is determined to be healed.

9. Social Harm Event (SHE): This form documents any social harm event experienced by the participants during the study.

10. Protocol Violated Form (PVF): This form describes any protocols violated during the study. This form is filled on the occurrence of PV

11. Final status form (FINAL): This form is completed to document when the participant leaves the study (i.e., completes follow-up, is discontinued, is declared lost to follow-up)

12. Query Response (QUERY). This form is filled to make corrections in the database.

The key features of the eCRFs are:

- Data validation checks
- Programmed skip patterns
- Pages customized according to individuals' tasks (e.g., nurse, doctor, etc.)
- Limits (e.g., text, numbers)
- Radio buttons (i.e., prompts to trigger clinicians to ask or look for specific things)

The eCRFs will undergo a series of tests before data collection to ensure they are all correct prior to the start of actual data collection.

1.4 Data collection using the eCRFs

A high level of confidentiality will be strictly adhered to in handling study data from the sites. The site's unique identification code and participant number will be used to identify each record in an electronic format. The study team will also use in-build provider identifiers for each record to enhance the audit trail of data if need be. The team at the study site will only enter study data for the participants.

Site data will be collected using Open Data Kit (ODK) and a computer tablet. ODK allows for 256-bit encrypted data collection forms, a mechanism for keeping data private after collection and during transit from the respective facilities to a secure server. Encrypted forms apply asymmetric public key encryption at the time the form is finalized within the ODK Collect module. This encrypted form can then be submitted up to the ODK Aggregate module and downloaded to the ODK Briefcase module. ODK Briefcase, when supplied with the asymmetric private key (which ODK Briefcase never stores), can then decrypt and export the form data as a CSV file for analysis and uploading to other servers by key study personnel.

ODK Aggregate cannot meaningfully publish encrypted forms to Google Spreadsheets or Fusion Tables since the encryption obscures the entire contents of the form, and ODK Aggregate never possesses the asymmetric key required to decrypt the form. When using encrypted forms, ODK Aggregate serves only as a data aggregation point — one must download, decrypt, and export the data using ODK Briefcase to access the unencrypted data.

All data collected using the tablet will be coded by Participant Unique ID and will not be accessible to anyone not affiliated with the research project. The field staff will not be able to access the completed interview file from the tablet. These security features will be utilized in order to provide the highest assurances to study participants that their responses will not later be available to the field study staff, and can only be accessed in the central study database by authorized personnel using the asymmetric private key.

Standardized Operating Procedures (SOPs) with resources and mechanisms for data synchronization to the aggregate server, cleaning/editing, and data entry preparation will be used to review data consistency checks and maintain the quality and integrity of the data accumulated. Quality assurance will be supported by examining patterns of missing data to correct any problems found.

1.4.1 Correcting data uploaded to the aggregate server

Data stored in the ODK Aggregate server is read-only and cannot be changed. Measures will be taken to ensure an audit trail of all changes made to the exported eCRFs. A Query Response eCRF will be filled by site study staff or any other authorized person to document changes made to the already uploaded data. The Query Response CRF (Annex 1) will have the participant's ID updated in the format "SFC/DDMMYY/Participant ID" input.

1.4.2 Data security

To enhance the level of data security, the ODK Aggregate server will have restricted access. A "Data Collect" account will be configured on the tablets used for data collection. This account has restricted rights on the Aggregate server and can only be used to upload collected data. Exported eCRFs will be downloaded to a USB drive and physically stored in a secure cabinet. All tablets used to collect data will have an access code/pattern to restrict access by unauthorized users. Transmission of data form the tablet to the Aggregate server will be over an encrypted connection over the internet.

1.5 Data confidentiality

All study staff will adhere to the highest level of confidentiality when handling study data as per the study protocol and the relevant code of ethics in research studies. All data exchange on e-mail will be password protected.

1.6 Data backup

A weekly, monthly, and completed study period of data backup plan will be enforced. The cloud real-timebdata backup will be performed daily and also save it on a USB drive, then data backup will be saved on CDROM drives. The backup storage will adhere to data security standards.

1.7 Summary of Analysis Strategy

Overall analysis strategy	Majorly descriptive	The analysis will be done to establish outcomes of each procedure (ShangRing and Mogen Clamp) and the relationship to the study site.
Study population	Treatment group	The analysis will ensure that the comparison of the results obtained from the treatment groups is obtained. The two groups are the ShangRing and Mogen clamp (control arm)
	Study site	The analysis will capture the specific outcomes of each of the sites in the three countries (Kenya, Tanzania, and Uganda).
Missing Data		-Queries will be sent to the site when missing data are identified in the database. To the extent, possible corrections to the database will be made before it is closed.<br>-No missing data will be imputed.

Inclusion/exclusion criteria	Inclusion	-As defined by the study protocol<br>-All clients screened will form initial analysis to ascertain the eligibility of clients<br>-The final analysis will encompass clients satisfying inclusion criteria who were randomized.
	Exclusion criteria	-As defined by the protocol<br>-the analysis will highlight any case where there was a deviation from the study protocol.
Reporting conventions	Data analysis and reporting	-Absolute numbers will be reported: count, means, standard deviations, maximum, and minimum.<br>-Proportions will be reported to one decimal place.
	Test of significance	-Continuous data will be compared using a two-sample t-test<br>-Proportions will be compared using a chi-square test, or Fisher's exact test of the assumptions of the chi-square test are violated
	Outliers	-The use of data validation in data entry into eCRFs will reduce incidences of outliers.<br>-Outliers identified in the database will be verified by sending queries to the study sites.

1.7.1 Final analysis

The final analysis and a report will be prepared upon completion of Phase 1 and will encompass all the indicators captured in the CRFs for those participants randomized during the study. The CSV files for each form will be read into R and then analysed. Table shells and listings are in Section 4.

The analysis will encompass all the quantitative data and qualitative data captured through the CRFs, which will include but not be limited to:

Demographic Data: Analysis will be done of the age, ethnic groups, level of education, and religion, etc. of the study participants

Medical history / physical exam: Analysis will be done for the participants' temperature, blood pressure (BP), the heartbeats per minute (BPM), height (ht), weight (wt) etc. at specific time intervals.

Summary of circumcision procedure: The analysis will be done for procedure time, amount of anaesthesia used, and ring sizes that were used. Problems encountered during the circumcision procedures will be descriptively summarized in a listing.

ShangRing removal and detachment: The average time taken during ring removal will be calculated. Numbers of participants with problems encountered during the removal procedures will be reported with specifics descriptively summarized in a listing. The degree of detachment of the ring from the skin will be analyzed. Issues around spontaneous detachment will include mean and median time to spontaneous detachment, the association between age and time to spontaneous detachment, the association between the comfort of clients and mode of detachment, experience/satisfaction among participants in the two groups, including requested early removal.

Adverse Events: For safety, clinical observations for adverse events during the course of wound healing will be analyzed. Adverse events will be reviewed on an ongoing basis during the study to determine any patterns in type, severity etc. that would suggest a problem that needs to be addressed. It will also indicate if the study

should be stopped due to safety concerns. The frequency and percentage of men with procedure-related AEs will be tabulated by severity, both overall and by the site. Listings of AEs will include information on duration, outcome, severity, and relation to study procedures. Listings of any serious adverse events will also be provided.

2.0 Final analysis table shells

2.1 Enrollment & final status

1a. Enrollment			
	**ShangRing n (%)**	**Mogen Clamp n (%)**	**Total**
Enrolled patients			
Enrolment by country			
1. UGANDA			
2. TANZANIA			
3. KENYA			

Reasons participants not enrolled by site:		
	**Reason***	**n (%)**
Reasons participants not enrolled by randomization group:		
	Reason	N (%)
ShangRing		
Mogen Clamp		

*Each patient was assigned one reason only. The reason assigned was the first reached in the list.

1b. Final study status grand totals – intention to treat population, n (%)				Comments
	ShangRing	Mogen Clamp	Total	
Discontinued				
Lost to follow-up				

Lost before ring removal or detachment documented				
Completed study [1]				
Healed before or at 42 days				
Required additional FU beyond 42 days				
Total				

[1]attended follow-up through documented healing or 42 days.

1c. Intent-to-treat population, n (%)			
	ShangRing	Mogen Clamp	Total
**Final study status**			
Discontinued			
Lost to follow-up			
Lost before ring removal or detachment documented			
Completed study [1]			
Healed before or at 42 days			
Required additional FU beyond 42 days			
Total			
Study visit completed			
Day 7			
Day 14			
Day 21			
Day 28			
Day 35			
Day 42			
Post-study Visit Day 49			
Post-study Visit Day 56			
Post-study visit day 63			
Unscheduled visits[3]			
0			
1			
2 (etc as needed)			
Total			

[1]attended follow-up through documented healing or 42 days.

1d. Reason for Unscheduled Visit (from MPFU), n (%)			
	ShangRing	Mogen Clamp	Total
AE			
The client requested ring removal			
Other (specify)			

1e. Participant's reason for withdrawal or discontinuation			
	ShangRing	Mogen Clamp	Total
Not eligible (screening failure)			
Specify Reason			
Eligible, not enrolled			
Specify Reason			
Discontinued/withdrawal			
Specify Reason			

2.2 Demographic Information

2a. Age (days)			
	ShangRing	Mogen Clamp	Total
Mean (SD)			
Median (Interquartile Range)			
Range (Min to Max)			
Age Group:			
≤ 7 days			
8-14 days			
15-21 days			
22-28 days			
29-35 days			
36-42 days			
43-49 days			
50-56 days			
57-60 days			

2b. Relationship of the participant to parent/LAR, n (%)			
	ShangRing	Mogen Clamp	Total
Father			
Mother			
Sibling			
Guardian			
Other (specify)			

2c. Age of Parent/LAR (days)			
	ShangRing	Mogen Clamp	Total
Mean (SD)			
Median (Interquartile Range)			
Range (Min to Max)			

2d. Marital Status			
	ShangRing	Mogen Clamp	Total
Cohabiting			
Divorced			
Married			
Single, never married			
Single, separated			
Other			

2e. Parent/LAR's highest level of academic education, n (%)			
	ShangRing	Mogen Clamp	Total
No formal education			
Some Primary			
Completed Primary			
Some Secondary			
Completed Secondary			
Some University/College/ Polytechnic			
Completed University/College/ Polytechnic			
Postgraduate			
Other, specify			

2f. Parent/LAR's religion, n (%)			
	ShangRing	Mogen Clamp	Total
Roman Catholic			
Anglican			
Pentecostal			
Seventh-Day Adventist			
Muslim			
Hindu			
Nomiya			
African Independent Church			
No Religion			
Other (Specify)			

2g. Ethnic Group of the parent/LAR, n (%)			
	ShangRing	Mogen Clamp	Total
Luo			
Kisii			
Luhya			
Teso			
Giriama			
Taita			
Kikuyu			
Kamba			
Other Specify			

2h. Primary Reasons for parent/LAR seeking circumcision for client, n (%)			
	ShangRing	Mogen Clamp	Total
HIV Protection			
Hygiene			
As part of medical therapy			
Social/religious			
Cultural			
Other (specify)			
Blank			

2.3 Intake

2.3.1 Client's HIV Status

3a. HIV status, n (%)			
	ShangRing	Mogen Clamp	Total
HIV negative			
HIV positive			
Tested but does not want to disclose			
Not tested			

2.3.2 Vital Signs & Weight

3b. Vital signs			
	ShangRing	Mogen Clamp	Total
Heart rate (beats/Min)			
Mean (SD)			
Min-Max			
Temperature (°C)			
Mean (SD)			
Min-Max			
Height (cm)			
Mean (SD)			
Min-Max			
Weight (Kgs)			
Mean (SD)			
Min-Max			
Respiratory rate (breaths/min)			
Mean (SD)			
Min-Max			

2.3.3 Client History

3c. Site of delivery for infant, n (%)			
	ShangRing	Mogen Clamp	Total
Home			
Facility			
Other (specify)			

3d. Mode of delivery, n (%)			
	ShangRing	Mogen Clamp	Total
Spontaneous vaginal delivery			
Assisted delivery (specify)			
Caesarean section			

3e. Parent has history of or undergoing treatment for illnesses, n (%)			
	ShangRing	Mogen Clamp	Total
Hemophilia or other bleeding disorder			
Diabetes			
Anemia			
Sickle cell			
Other (specify)			

3f. Current conditions: Infant presenting with any of the listed conditions/complaints, n (%)			
	ShangRing	Mogen Clamp	Total
Fever			
Genital sore (ulcer)			
Jaundice			
Urethral discharge			
Swelling of the scrotum			
Other (specified)			

3g. Participants have any known allergies, n (%)			
	ShangRing	Mogen Clamp	Total
Yes			
No			

3h. Known allergy description					
Country	Study Arm	Allergy description	Manifestation	Treatment if known	n

3i. Participants ever had the surgical operation, n (%)			
	ShangRing	Mogen Clamp	Total
Yes (specify)			
No			

2.4 Eligibility for Circumcision & Study

4a. Inclusion/Exclusion criteria, n (%)			
	ShangRing	Mogen Clamp	Total
**INCLUSION (all should be 100%)**			
60 days or younger			
birth weight ≥2.5kg			
gestational age ≥37 weeks			
uncircumcised with a fully intact foreskin			
in good general health			
free of genital ulcerations/infections			
parent(s)/LAR understand all study procedures and requirements			
parent(s)/LAR agree to return the participant for all follow-up visits			
parent(s)/LAR have a cellphone or access to a cellphone			
parent(s)/LAR agree to provide an address, phone number or other locator information while participating in the study			
**EXCLUSION (all should be 0%)**			
Have known allergy to lidocaine/other local anesthesia			
Have an active genital infection, anatomic abnormality or other condition that prevents him from undergoing a circumcision			
On blood thinners, steroids, or other medications that may preclude him			

from normal wound healing/have a family history of bleeding disorders			
Have a condition that contradicts participation in the study			
currently participating in another biomedical research study			

4b. Eligibility			
	ShangRing	Mogen Clamp	Total
Eligible for study, n (%)			
Reasons for those not eligible<br>Reason 1<br>Reason 2<br>Reason 3			
Otherwise medically eligible for ShangRing circumcision, n (%)			
Reasons for those not eligible<br>Reason 1<br>Reason 2<br>Reason 3			
Would be eligible for conventional circumcision, n (%)			
Reasons for those not eligible<br>Reason 1<br>Reason 2<br>Reason 3			

2.5 Operation

5a. Was there need to supplement topical anesthesia with injectable anesthesia before the start of the procedure?			
	ShangRing	Mogen Clamp	Total
Yes			
No			

5b. NIPS pain score immediately prior to circumcision				
		ShangRing	Mogen Clamp	Total
Facial expression	Relaxed			
	Grimacing			
Cry	No Cry			
	Whimper			
	Vigorous cry			
Breathing Pattern	Relaxed			
	Change in breathing			
Arms	Restrained or Relaxed			
	Flexed or Extended			
Legs	Restrained or Relaxed			
	Flexed or Extended			
State of arousal	Sleeping or Awake			
	Fussy			
Total NIPS score				
Mean (SD)				
Min-Max				

5c. Adhesions, n (%)			
	ShangRing	Mogen Clamp	Total
Necessary to break down adhesions between the foreskin and head of the penis			
Degree of adhesions (among those where adhesions has to be broken down)			
Mild			
Moderate			
Severe			

5d. Circumcision procedure duration in minutes			
	ShangRing	Mogen Clamp	Total
Mean duration (in min)			
SD			
Median (IQR)			
Max			
Min			
Outliers			

5e. Type of circumcision procedure conducted, n (%)			
	ShangRing	Mogen Clamp	Total
ShangRing			
Mogen clamp			
Other (specified)			
If Other, please explain:			

5f. ShangRing Only:	
Need for dorsal slit in the foreskin in order to insert ring, n (%)	ShangRing Group Only
Yes	
No	
Mean length of dorsal slit (in cm)	
SD	
Max	
Min	
Outliers	
Ring Size, n (%)	
X	
Y	
Z	

5g. Ring Size by Country			
Ring Size, n (%)	Uganda	Kenya	Tanzania
X			
Y			
Z			

5h. Pain score during circumcision using the RNCS				
Step		ShangRing	Mogen Clamp	Total
Placement of hemostats	No cry (0)			
	Whimper (0.5)			
	Vigorous cry (1)			
Separation of adhesions	No cry (0)			
	Whimper (0.5)			
	Vigorous cry (1)			
Retraction & reduction of prepuce	No cry (0)			
	Whimper (0.5)			
	Vigorous cry (1)			
Dorsal slit OR Placement of device (Mogen Clamp)	No cry (0)			
	Whimper (0.5)			
	Vigorous cry (1)			
Placement of inner ring OR Activation of the device (Mogen Clamp)	No cry (0)			
	Whimper (0.5)			
	Vigorous cry (1)			
Placement of outer ring (first click) OR Deactivation and removal of the device (Mogen Clamp)	No cry (0)			
	Whimper (0.5)			
	Vigorous cry (1)			
Skin adjustment with the ring in place OR Delivery of glans (Mogen Clamp)	No cry (0)			
	Whimper (0.5)			
	Vigorous cry (1)			
Locking of the outer ring (second click) OR Vaseline application (Mogen Clamp)	No cry (0)			
	Whimper (0.5)			
	Vigorous cry (1)			
Excision of foreskin OR Dressing with gauze and diaper (Mogen Clamp)	No cry (0)			
	Whimper (0.5)			
	Vigorous cry (1)			
**Total**				
Mean (SD)				
Min-Max				

5i. NIPS pain score during circumcision				
		ShangRing	Mogen Clamp	Total
Facial expression	Relaxed			
	Grimacing			
Cry	No Cry			
	Whimper			
	Vigorous cry			
Breathing Pattern	Relaxed			
	Change in breathing			
Arms	Restrained or Relaxed			
	Flexed or Extended			
Legs	Restrained or Relaxed			
	Flexed or Extended			
State of arousal	Sleeping or Awake			
	Fussy			
Total NIPS score				
Mean (SD)				
Min-Max				

5j. Anaesthesia Information, n (%)			
	ShangRing	Mogen Clamp	Total
Topical anaesthetic successfully used for circumcision			
Yes			
No			
If no, was there need to supplement with injectable anaesthesia after the procedure began?			
Yes			
No			
Did the infant have a reaction to the anaesthesia			
Yes			
No			
Was any medication given to the client before the surgical procedure was conducted?			
Yes			
No			

5k. Anesthetic dwell time*			
	ShangRing	Mogen Clamp	Total
Mean duration (in min)			
SD			
Max			
Min			
Outliers			

*It is important to keep in mind that dwell time was not an accurate reflection of the time it took for the anaesthetic to take effect, as the providers would check on the participants at intervals, and at times had to finish attending to other duties before coming back to check on the participant.

5l. Were there any problems or difficulties with the circumcision procedure?				
By Site		ShangRing	Mogen Clamp	Total
1. HOMA BAY	Yes			
	No			
2. KISUMU	Yes			
	No			
3. KAKUUTO	Yes			
	No			
4. KALISIZO	Yes			
	No			
5. LYANTONDE	Yes			
	No			
6. MASAKA	Yes			
	No			
7. RAKAI	Yes			
	No			
8. NGOME HEALTH CENTER	Yes			
	No			
9. IRINGA REGIONALHOSPITAL	Yes			
	No			
10. MAFINGA DISTRICT HOSPITAL	Yes			
	No			
11. ILULA HOSPITAL	Yes			
	No			

If yes describe				
Participant number(s)	Country	Study Arm	Description of the problem or difficulty	Frequency

5m. The cadre of MC provider performing the MC			
	ShangRing	Mogen Clamp	Total
Doctor			
Clinical Officer			
Nurse			

5n. Cadre of MC assistant			
	ShangRing	Mogen Clamp	Total
Doctor			
Clinical Officer			
Nurse			

2.6 Post Operation

6a. NIPS pain score around 20 minutes post op, n (%)				
		ShangRing	Mogen Clamp	Total
Facial expression	Relaxed			
	Grimacing			
Cry	No Cry			
	Whimper			
	Vigorous cry			
Breathing Pattern	Relaxed			
	Change in breathing			
Arms	Restrained or Relaxed			
	Flexed or Extended			
Legs	Restrained or Relaxed			
	Flexed or Extended			
State of arousal	Sleeping or Awake			
	Fussy			
Total NIPS score				
Mean (SD)				
Min-Max				

6b. Average time after operation was the pain recorded			
	ShangRing	Mogen Clamp	Total
Mean			
SD			
Median			
Min			
Max			

6c. Status of the circumcision wound before discharge					
			ShangRing	Mogen Clamp	Total
Normal					
If Abnormal, please describe					
Participant number(s)	Study Arm	Country	Site	Description	Frequency n

6d. Medication given during post-op period prior to discharge				
		ShangRing	Mogen Clamp	Total
Any medication other than paracetamol given to the client to manage postoperative discomfort: Yes, n (%)				
If yes, state dosage				
Participant number(s)	Study Arm	Country	Site	Description

2.7 Follow-up

2.7.1 24hr Call Interview

7a. 24hr call interview information, n (%)			
	ShangRing	Mogen Clamp	Total
Was the phone call made successfully?			
Yes			
No			
Is the Baby well?			
Yes			
No			

7b. Listing of issues reported if the baby is not well:

Participant #	Study Arm	Country	Site	Comments

2.7.2 Genital Examination

7c. Overall summary: Aside from the circumcision wound, are genitals normal			
	ShangRing	Mogen Clamp	Total
7-day visit			
14-day visit			
21-day visit			

28-day visit			
35-day visit			
42-day visit			

7d. Listing of abnormalities reported:

Participant #	Study Arm	Country	Site	Visit day	Abnormality

2.7.3 Wound Assessment (ShangRing Only)

7e. Wound Assessment

	ShangRing Group Only						
Visit Day	Day 7	Day 14	Day 21	Day 28	Day 35	Day 42	Total
Is the ShangRing still in place ?							
Yes							
No							
Degree of separation between the ShangRing and the penis							
No detachment							
Partly detached							
Completely detached							
If detached, was the ShangRing returned to the clinic?							
Yes							
No							
Is ShangRing being removed today?							
Yes							
No							
Dressing applied after ShangRing removal							
Yes							
No							
Problems during ShangRing removal							
Yes							
No							
If yes above, explain:							

Participant number	Country	Site	Study Arm	Description

7f. Overall summary: Reasons for ShangRing removal							
Reasons for ShangRing removal	ShangRing						
	Day 7	Day 14	Day 21	Day 28	Day 35	Day 42	Total
Pain							
Swelling							
Infection							
Other (specify)							

7g. Staff Information							
	ShangRing Group Only						
Visit Day	Day 7	Day 14	Day 21	Day 28	Day 35	Day 42	Total
Number of staff assisted with the removal							
Mean (SD)							
Median (IQR)							
The cadre of the person responsible for removal, n (%)							
Physician							
VMMC medical provoder							

7h. NIPS pain score during ring removal								
		ShangRing Group Only						
		Day 7	Day 14	Day 21	Day 28	Day 35	Day 42	Total
Facial expression	Relaxed							
	Grimacing							
Cry	No Cry							
	Whimper							
	Vigorous cry							
Breathing Pattern	Relaxed							
	Change in breathing							
Arms	Restrained or Relaxed							
	Flexed or Extended							
Legs	Restrained or Relaxed							
	Flexed or Extended							
State of arousal	Sleeping or Awake							
	Fussy							
Total NIPS score								
Mean (SD)								
Min-Max								

7i. Medication given during follow-up visit, n (%)			
	ShangRing	Mogen clamp	Total, n (%)
Yes			
If yes, state dosage			

Participant number(s)	Arm	Country	Site	Duration	Name of drug	Indication	Doses	Route

2.7.4 Ring Removal

7j. Removals Grand Totals by site							
	ShangRing Group Only						
	Day 7	Day 14	Day 21	Day 28	Day 35	Day 42	Total
1. HOMA BAY							
2. KISUMU							
3. KAKUUTO							
4. KALISIZO							
5. LYANTONDE							
6. MASAKA							
7. RAKAI							
8. NGOME HEALTH CENTER							
9. IRINGA REGIONALHOSPITAL							
10. MAFINGA DISTRICT HOSPITAL							
11. ILULA HOSPITAL							

Notes on Removals in ShangRing Group

Participant #	Who requested (px or provider)	Reason noted & other details (i.e. he had an AE)

2.8 Healing information

8a. Visit day declared completely healed, n (%)			
	ShangRing	Mogen Clamp	Total
Day 14			
Day 21			
Day 28			
Day 35			
Day 42			
Day 49			
Unscheduled			
Beyond Day 49			

8b. Kaplan-Meier Estimates of the Cumulative Probability of Complete Healing, by Randomization Group								
	ShangRing				Mogen Clamp			
Day of Study Visit	No. Left[1]	Cum. No. Healed[2]	Cum. Prob. Healed	95% CI	No. Left[1]	Cum. No. Healed[2]	Cum. Prob. Healed	95% CI
7								
14								
21								
28								

8b. Kaplan-Meier Estimates of the Cumulative Probability of Complete Healing, by Randomization Group								
	ShangRing				Mogen Clamp			
35								
42								

1 The number of infants infants to attain complete healing and still being followed before each study visit.

2 The number of infants who attained complete healing by study visit.

8c. Time to Healing calculated in Days			
	ShangRing	Mogen Clamp	Total
Mean (SD)			
Median (IQR)			
Min			
Max			

2.9 Completion Interview

9a. Completion Interview Visit Day			
	ShangRing	Mogen Clamp	Total
7-day visit			
14-day visit			
21-day visit			
28-day visit			
35-day visit			
42-day visit			

9b. What was liked about the circumcision procedure (multiple responses possible)			
	ShangRing	Mogen Clamp	Total
Less pain than expected			
Circumcision was quick			
No dressing applied to wound			
Improved personal hygiene			
Cosmetic appearance			
No injection needed			
No stitches needed			
The device fell off on its own			
Other (explain)			

9c. What was not liked about the circumcision procedure (multiple responses possible)			
	ShangRing	Mogen Clamp	Total
More pain than expected			
Circumcision took long to heal			
Wound care during healing was difficult			
Not the best cosmetic appearance			
There was nothing I disliked about the circumcision			
Other (explain			

9d. How satisfied are you with the appearance of the healed circumcised penis? (			
	ShangRing	Mogen Clamp	Total
Participant's satisfaction with the appearance of the healed circumcised penis			
Very satisfied			
Somewhat satisfied			
Some dissatisfied			
Not satisfied at all			
Would you recommend circumcision at this age to another parent/LAR?			
Yes			
no			
If no, explain why:			

2.10 Adverse Events

2.10.1 Summary Data for all AEs

10a. Total AEs by study arm			
Study arm	ShangRing	Mogen Clamp	Total
Total number of AEs			
Mild			
Moderate			
Severe			
Serious			
Total number of participants with an AE			

10b. Total AEs by country and study arm						
Country	Uganda		Tanzania		Kenya	
Study arm	ShangRing	Mogen Clamp	ShangRing	Mogen Clamp	ShangRing	Mogen Clamp
Total number of AEs						
Mild						
Moderate						
Severe						
Serious						
Total number of participants with an AE						

10c. Total AEs by site and study arm			
Study arm	ShangRing	Mogen Clamp	Total
HOMA BAY			
KISUMU			
KAKUUTO			
KALISIZO			
LYANTONDE			
MASAKA			
RAKAI			
NGOME HEALTH CENTER			
IRINGA REGIONALHOSPITAL			
MAFINGA DISTRICT HOSPITAL			
ILULA HOSPITAL			

10d. How related is the AE to the circumcision procedure, by study arm			
Study Arm	ShangRing	Mogen Clamp	Total
Unrelated			
Possibly related			
Probably related			
Definitely related			

2.10.2 Summary Data for AEs during the procedure and post-operation

10e. Total AEs during circumcision procedure & post-op period (i.e. before discharge), by study arm			
Study Arm	ShangRing	Mogen Clamp	Total
Total number of AEs			
Mild			
Moderate			
Severe			
Serious			
Total number of participants with an AE			

10f. Total AEs during circumcision procedure & post-op period (i.e. before discharge), by country									
Country	Uganda			Tanzania			Kenya		
Study Arm	ShangRing	Mogen Clamp	Total	ShangRing	Mogen Clamp	Total	ShangRing	Mogen Clamp	Total
Total number of AEs									
Mild									
Moderate									
Severe									
Serious									
Total number of participants with an AE									

10g. How related is the postop AE to the circumcision procedure, by study arm			
Study Arm	ShangRing	Mogen Clamp	Total
Unrelated			
Possibly related			
Probably related			
Definitely related			

10h. How related is the postop AE to the circumcision procedure, by country and study arm									
	Uganda			Tanzania			Kenya		
Study Arm	ShangRing	Mogen Clamp	Total	ShangRing	Mogen Clamp	Total	ShangRing	Mogen Clamp	Total
Unrelated									
Possibly related									
Probably related									
Definitely related									

10i. Was the post-op AE treated?			
	ShangRing	Mogen Clamp	Total
Yes			
No			

If yes describe		
Participant ID	Study Arm	Description

10j. What action was taken regarding ShangRing device	
	ShangRing Group Only
None, device not present	
Device not removed	
Device removed	

10k. What action was taken regarding the ShangRing device (ShangRing Group Only) by Country			
	Uganda	Tanzania	Kenya
None, device not present			
Device not removed			
Device removed			

10l. Postop AE resulted in death (Y/N), by country and study arm									
	Uganda			Tanzania			Kenya		
	Shang Ring	Mogen Clamp	Total	Shang Ring	Mogen Clamp	Total	Shang Ring	Mogen Clamp	Total
Yes									
No									

Insert the AE listing here (should we just list moderate and severe?)

The listing should have these sorts of details:
Participant number #
Randomization group
Timing (intra op, post op or # days follow-up)
Type of AE
Severity (mild, moderate, severe)
Serious (yes or no)
Related (Not Related, Possibly Related, Probably Related, Definitely Related)
Action taken with ShangRing
How treated
Treatment
Medication given
Indication

Maybe it would be good to have subheading rows for each country as the country is not necessarily apparent from the participant number unless one remember what all the two letter codes are.

10m. AEs during the ShangRing/Mogen Clamp procedure and immediate post-operative period (before discharge)			
Study Arm	ShangRing	Mogen Clamp	Total
Total AEs			
Type of AE			
Excessive pain			
Moderate			
Severe			
Bleeding			
Moderate			
Severe			
Anaesthetic reaction			
Moderate			
Severe			
Severe NIPS pain			
Moderate			
Severe			
Other (specify)			
Moderate			
Severe			

10n. AEs during the ShangRing/Mogen Clamp procedure and immediate post-operative period (before discharge), by country and Study Arm									
	Uganda			Tanzania			Kenya		
Study Arm	ShangRing	Mogen Clamp	Total	ShangRing	Mogen Clamp	Total	ShangRing	Mogen Clamp	Total
Total AEs									
Type of AE									
Excessive pain									
Moderate									
Severe									
Bleeding									
Moderate									
Severe									
Anaesthetic reaction									
Moderate									
Severe									
Severe NIPS pain									
Moderate									
Severe									
Other (specify)									
Moderate									
Severe									

2.10.3 Summary Data for Follow-up AEs

10o. Total follow-up AEs, by study arm			
Study Arm	ShangRing	Mogen Clamp	Total
Total number of AEs			
Mild			
Moderate			
Severe			
Serious			
Total number of participants with an AE			

10p. Total follow-up AEs, by country and study arm									
Country	Uganda			Tanzania			Kenya		
Study Arm	ShangRing	Mogen Clamp	Total	ShangRing	Mogen Clamp	Total	ShangRing	Mogen Clamp	Total
Total number of AEs									
Mild									
Moderate									
Severe									
Serious									
Total number of participants with an AE									

10q. How related is the follow-up AE to the circumcision procedure, by study arm			
Study Arm	ShangRing	Mogen Clamp	Total
Unrelated			
Possibly related			
Probably related			
Definitely related			

10r. How related is the follow-up AE to the circumcision procedure, by country and study arm									
	Uganda			Tanzania			Kenya		
Study Arm	ShangRing	Mogen Clamp	Total	ShangRing	Mogen Clamp	Total	ShangRing	Mogen Clamp	Total
Unrelated									
Possibly related									
Probably related									
Definitely related									

10s. Was the follow-up AE treated?			
	ShangRing	Mogen Clamp	Total
Yes			
No			
If yes describe			
Participant ID	Study Arm	Description	

10t. What action was taken regarding ShangRing device	
	ShangRing Group Only
None, device not present	
Device not removed	
Device removed	

10u. What action was taken regarding the ShangRing device (ShangRing Group Only) by Country			
	Uganda	Tanzania	Kenya
None, device not present			
Device not removed			
Device removed			

10v. Follow-up AE resulted in death (Y/N), by country and study arm									
	Uganda			Tanzania			Kenya		
	Shang Ring	Mogen Clamp	Total	Shang Ring	Mogen Clamp	Total	Shang Ring	Mogen Clamp	Total
Yes									
No									

Insert the AE listing here (should we just list moderate and severe?)

The listing should have these sorts of details:
Participant number #
Randomization group
Timing (intra op, post op or # days follow-up)
Type of AE
Severity (mild, moderate, severe)
Serious (yes or no)
Related (Not Related, Possibly Related, Probably Related, Definitely Related)
Action taken with ShangRing
How treated
Treatment
Medication given
Indication

Maybe it would be good to have subheading rows for each country as the country is not necessarily apparent from the participant number unless one remember what all the two letter codes are.

10w. AEs during follow-up			
Study Arm	ShangRing	Mogen Clamp	Total
Total AEs			
Type of AE			
Hematoma			
Moderate			
Severe			
Swelling			
Moderate			
Severe			
Torsion			
Moderate			
Severe			
Cutaneous pinch			
Moderate			
Severe			
Difficulty urinating			
Moderate			
Severe			
Pain with erections			
Moderate			
Severe			
Insufficient skin removal			
Moderate			
Severe			
Buried penis			
Moderate			
Severe			

10w. AEs during follow-up			
Study Arm	ShangRing	Mogen Clamp	Total
Severe NIPS pain			
Moderate			
Severe			
Other (specify)			
Moderate			
Severe			

10x. AEs during follow up, by country									
Country	Uganda			Kenya			Tanzania		
Study Arm	Shang Ring	Mogen Clamp	Total	Shang Ring	Mogen Clamp	Total	Shang Ring	Mogen Clamp	Total
Total AEs									
Type of AE									
Hematoma									
Moderate									
Severe									
Swelling									
Moderate									
Severe									
Torsion									
Moderate									
Severe									
Cutaneous pinch									
Moderate									
Severe									
Difficulty urinating									
Moderate									
Severe									
Pain with erections									
Moderate									
Severe									
Buried penis									
Moderate									
Severe									
Severe NIPS pain									

10x. AEs during follow up, by country									
Country	Uganda			Kenya			Tanzania		
Study Arm	Shang Ring	Mogen Clamp	Total	Shang Ring	Mogen Clamp	Total	Shang Ring	Mogen Clamp	Total
Moderate									
Severe									
Other (specify)									
Moderate									
Severe									

6.0Annex

Annex 1: Query Response CRF

Below are the questions that were included in the form. Like the other CRFs, an electronic version was used.

1. **The date this correction is being made?{read only}**
   DD/MM/YYYY

2. **Client ID of the eCRF in which the change is being made?.**

3. **Type of eCRF the correction is being made?**
   Demo
   Intake & Screening
   OPR
   POST-OPR
   MASTER PFU [GOTO 3a]
   IVW
   FINAL STATUS

3a. **what is the visit day of the Master PFU the correction being made?**
   Day 7
   Day 14
   Day 21
   Day 28
   Day 35
   Day 42
   Unscheduled

4. **What is the question that is being corrected? E.g. 1, 2a, 3, 4b etc..**

5. **The previous value entered?**

6. **New value entered**

7. **Is there another correction that is being made on this eCRF?**
   Yes [Repeat Questions 4, 5, 6 and & 7 until response to question 7 = NO]

   No

Supplementary Table. Moderate and severe adverse events based on WHO/JHPIEGO definitions.

Adverse Event	Moderate	Severe
Bleeding	**Tx:** pressure dressing ± skin sutures	**Tx:** transfusion, surgical exploration, hospitalization ± transfer to another facility
Infection	Discharge, painful swelling/erythema, or fever<br>**Tx:** enteral antibiotics only	Severe infection, cellulitis, abscess<br><br>**Tx:** surgical intervention, hospitalization, parenteral antibiotics
Wound disruption	Defect >1.0 cm in length<br>**Tx:** suturing or other clinical intervention but not surgery	Any defect that requires surgical repair or referral/transfer to another facility
Pain	NIPS score of 3-5	NIPS score of 5 or greater
Penile scarring	Discernable but not requiring re-operation	Discernable and requires re-operation
Penile torsion	Torsion causing mild pain with erections, not requiring surgery	Painful erections requiring surgical correction
Insufficient skin removal	Prepuce partially covers glans when flaccid, not requiring surgery	Prepuce covers most of the glans when flaccid requiring surgical correction
Excess skin removal	Tightness of the skin + sutures or skin mobilization needed for closure<br><br>Pulling of scrotal skin onto penile shaft<br>Wound/suture disruption due to tension on stitches	Provider unable to close skin requiring referral to another facility<br><br>Gaping edges, large or deep defects that would lead to scar without revision
Penile injury (Abrasion, laceration, burn, urethral injury)	Prolonged intra-operative attention or pressure dressing<br><br>Prolonged follow-up care ± frequent dressings but not requiring surgery	• Severe laceration<br>• Severing of the glans or shaft<br>• Significant diathermy (+ /- tissue necrosis/loss)<br>• Urethral fistula development (delayed complication)<br><br>**Tx:** Additional surgeries, hospitalization, transfer or transfusion
Excess swelling, hematoma	Requires only clinical intervention	Surgical exploration required to control bleeding or removal of hematoma ± disability ≥8 days ± device placement or removal.
Voiding issue	Requires unscheduled clinic visit but no catheter placement	**Tx:** Catheter placement, treatment referral or surgery to correct.
